# Endoscopic Submucosal Dissection Versus Laparoscopic and Endoscopic Cooperative Surgery for Superficial Duodenal Epithelial Tumors: A Multicenter Retrospective Study

**DOI:** 10.1002/ags3.70056

**Published:** 2025-06-23

**Authors:** Takuo Takehana, Tsuneo Oyama, Motohiko Kato, Shunsuke Yoshii, Shu Hoteya, Satoru Nonaka, Shoichi Yoshimizu, Masao Yoshida, Ken Ohata, Hironori Yamamoto, Yuko Hara, Shigetsugu Tsuji, Osamu Dohi, Yasushi Yamasaki, Hiroya Ueyama, Koichi Kurahara, Tomoaki Tashima, Nobutsugu Abe, Atsushi Nakayama, Ichiro Oda, Naohisa Yahagi

**Affiliations:** ^1^ Department of Gastroenterological Surgery Saku Central Hospital Advanced Care Center Nagano Japan; ^2^ Department of Endoscopy Saku Central Hospital Advanced Care Center Nagano Japan; ^3^ Center for Diagnostic and Therapeutic Endoscopy Keio University School of Medicine Tokyo Japan; ^4^ Department of Gastrointestinal Oncology Osaka International Cancer Institute Osaka Japan; ^5^ Department of Gastroenterology Toranomon Hospital Tokyo Japan; ^6^ Endoscopy Division National Cancer Center Hospital Tokyo Japan; ^7^ Department of Gastroenterology Cancer Institute Hospital of the Japanese Foundation for Cancer Research Tokyo Japan; ^8^ Division of Endoscopy Shizuoka Cancer Center Shizuoka Japan; ^9^ Department of Gastrointestinal Endoscopy NTT Medical Center Tokyo Tokyo Japan; ^10^ Department of Medicine, Division of Gastroenterology Jichi Medical University Tochigi Japan; ^11^ Department of Endoscopy The Jikei University School of Medicine Tokyo Japan; ^12^ Department of Gastroenterology Ishikawa Prefectural Central Hospital Kanazawa Japan; ^13^ Molecular Gastroenterology and Hepatology, Graduate School of Medical Science Kyoto Prefectural University of Medicine Kyoto Japan; ^14^ Department of Gastroenterology Okayama University Hospital Okayama Japan; ^15^ Department of Gastroenterology Juntendo University School of Medicine Tokyo Japan; ^16^ Division of Gastroenterology Matsuyama Red Cross Hospital Matsuyama Japan; ^17^ Department of Gastroenterology Saitama Medical University International Medical Center Saitama Japan; ^18^ Department of Gastroenterological and General Surgery Kyorin University School of Medicine Tokyo Japan; ^19^ Division of Research and Development for Minimally Invasive Treatment, Cancer Center Keio University School of Medicine Tokyo Japan

**Keywords:** adverse event, endoscopic submucosal dissection, laparoscopic and endoscopic cooperative surgery, superficial duodenal epithelial tumor

## Abstract

**Aims:**

This study aimed to compare the clinicopathological features and short‐term outcomes of endoscopic submucosal dissection (ESD) and laparoscopic and endoscopic cooperative surgery (LECS) for superficial duodenal epithelial tumors (SDETs) and investigate the risk factors for severe adverse events (AEs).

**Methods:**

Overall, 1017 patients who underwent ESD and 62 who underwent LECS to treat suspected SDETs between January 2008 and December 2018 were included. After comparing surgical and postsurgical outcomes between the two groups, logistic regression analyses were performed to identify the predictors of AEs.

**Results:**

The lesion size was significantly larger in the LECS group than in the ESD group (28.5 vs. 20.8 mm, *p* < 0.01). The LECS group included significantly more patients with lesions greater than half the circumference than did the ESD group (19.3% vs. 5.4%, *p* < 0.01). LECS achieved a significantly higher complete closure rate of the resected wounds (98.4% vs. 74.4%, *p* < 0.01). Delayed bleeding and perforation occurred in 49 (5.0%) and 22 (2.2%) patients in the ESD group and 3 (4.8%) and 1 (1.6%) in the LECS group, respectively. Multivariate analyses revealed that incomplete closure of the resected wounds was the only independent risk factor for delayed bleeding (odds ratio, 5.069) and delayed perforation (odds ratio, 5.413).

**Conclusions:**

Both ESD and LECS showed similar AE rates, although LECS is likely to be indicated for larger and wider circumferential tumors. Only incomplete closure of the resected wound was an independent risk factor for severe AEs, such as delayed perforation and bleeding.

## 1 Introduction

The incidence of superficial duodenal epithelial tumors (SDETs) was previously considered extremely low [1, 2, 3]. However, the detection rate of SDETs has been increasing in recent years owing to advancements in endoscopic technologies and routine observation of the duodenum during screening and surveillance endoscopy [4, 5]. Surgical treatments such as pancreaticoduodenectomy were previously performed for these lesions. To preserve organ function as well as maintain patients' post‐treatment quality of life, various endoscopic resection (ER) techniques are now widely applied for SDETs, including cold snare polypectomy, endoscopic mucosal resection (EMR), underwater EMR, and endoscopic submucosal dissection (ESD). Among these, ESD achieved a higher rate of *en bloc* resection and R0 resection than other ER techniques, regardless of the lesion size [6]. However, ESD is technically demanding and occasionally causes severe adverse events (AEs) such as delayed bleeding and perforation [7, 8, 9, 10, 11, 12, 13, 14]. Delayed duodenal perforation can cause potentially fatal peritonitis due to biliary and pancreatic leakage, potentially necessitating surgical interventions such as pancreaticoduodenectomy.

Laparoscopic and endoscopic cooperative surgery (LECS) has been developed for gastric subepithelial tumors as a new minimally invasive surgery [15, 16] and has recently been applied to duodenal tumors to compensate for the weakness of duodenal ESD and prevent severe AEs [17]. Although the feasibility and safety of duodenal LECS have been reported previously [18, 19, 20, 21, 22], most of those studies were based on very limited single‐institutional experience, and few reports were available comparing post‐therapeutic outcomes between ESD and LECS for SDETs [23].

We have previously reported the outcomes of ER for SDETs, wherein LECS cases were excluded from the analyses [24]. In the present study, we compared the clinicopathological features and short‐term outcomes of patients between two promising treatment options, ESD and LECS, and investigated the risk factors for severe post‐therapeutic AEs using both techniques.

## 2 Methods

This study was a supplementary analysis of our previously published study [24]. This multicenter, retrospective, observational study was conducted at 18 institutions throughout Japan. A total of 3172 patients underwent ER between January 2008 and December 2018 for the treatment of suspected SDETs. Of these, 1017 patients who underwent ESD and 62 who underwent LECS were included in this study. The study protocol was approved by the Institutional Review Board of Keio University School of Medicine on October 28, 2019 (approval no. 20180288) and then by each institution. Informed consent was obtained in an opt‐out manner on the website of each institution. This study was conducted in accordance with the principles of the Declaration of Helsinki and its amendments. Treatment selection between ESD and LECS was determined collaboratively by endoscopists and surgeons at each institution, based on location, circumferential orientation, lesion size, circumferential extent, and technical feasibility. No standardized institutional protocol or unified selection criteria were established during the study period. There are two types of LECS: one method involves performing ESD followed by laparoscopic suturing and reinforcement of the resected wound (LECS‐ESD), and the other involves laparoscopic suturing and closure after full‐thickness resection (LECS‐FTR). Complete closure of the resected wound was defined as endoscopically confirmed full closure of the mucosal defect, with no exposure of the submucosal layer. For ESD cases, various closure techniques were used depending on the institution and lesion characteristics, including simple clip closure, an endoloop/clipping technique, the string clip suturing method, and over‐the‐scope (OTS) clips. In LECS cases, closure was mainly performed laparoscopically, either by suturing or reinforcement of the resected wound. The choice of closure technique was not standardized across institutions. First, we compared surgical and postsurgical outcomes between the ESD and LECS groups. Subsequently, we performed logistic regression analyses to identify predictors of delayed bleeding and perforation among various clinical characteristics (age, location, circumferential orientation, lesion size, circumferential extent, and macroscopic type) and treatment factors (treatment method, procedure time, and degree of closure). Statistical analyses were performed using JMP software (SAS Institute, Cary, NC, USA), and a *p* < 0.05 was considered statistically significant.

## 3 Results

### 3.1 Patients

**FIGURE 1:**
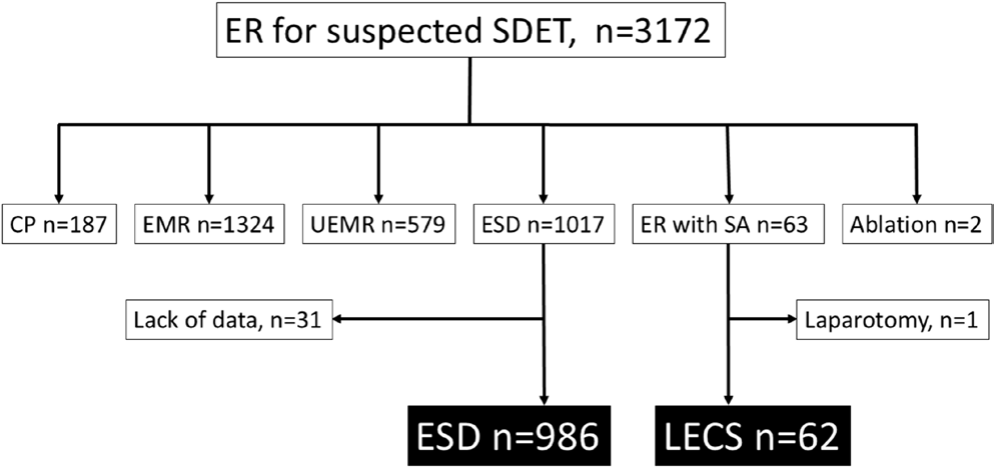
Flow‐chart of the study population. ER, endoscopic resection; SDET, superficial duodenal epithelial tumor; CP, cold polypectomy; EMR, endoscopic mucosal resection; UEMR, underwater EMR; ESD, endoscopic submucosal dissection; SA, surgical assistance; LECS, laparoscopic and endoscopic cooperative surgery.

Among the 1017 patients who underwent ESD, 31 were excluded from the ESD group owing to a lack of data. Therefore, 986 and 62 patients who underwent ESD and LECS, respectively, were included in the comparative analysis (Figure 1). Of the 62 patients with LECS, 48 (77.4%) underwent LECS‐ESD, 13 (21.0%) underwent LECS‐FTR, and 1 (1.6%) case was converted to open surgery.

### 3.2 Clinicopathological Characteristics

The clinical and pathological characteristics of patients who underwent ESD and LECS are shown in Table 1. The lesion size was significantly larger in the LECS group than in the ESD group (28.5 vs. 20.8 mm, *p* < 0.01). The location of the lesion did not differ between the two groups; however, the LECS group included significantly more patients with lesions greater than half the circumference than did the ESD group (19.3% vs. 5.4%, *p* < 0.01). The final pathological diagnosis revealed that the LECS group had a higher proportion of intramucosal and submucosal carcinomas than did the ESD group (75.8% vs. 52.1% and 4.8% vs. 2.8%, respectively). However, there were no significant differences in the other clinical characteristics between the two groups.

**TABLE 1:** Clinicopathological characteristics of patients who underwent ESD and LECS.

	ESD (*n* = 986)	LECS (*n* = 62)	*p*
Age, mean (SD), years	62.5 (11.0)	63.8 (11.3)	0.30
Sex, male, *n* (%)	669 (61.8%)	36 (58.1%)	0.11
Location			
Bulbs	134 (13.6%)	6 (9.7%)	
Supraduodenal angle	104 (10.6%)	6 (9.7%)	
Descending part oral	235 (23.8%)	18 (29.0%)	
Descending part anal	363 (36.8%)	20 (32.2%)	
Infraduodenal angle	96 (9.7%)	7 (11.3%)	
Horizontal part	53 (5.4%)	5 (8.1%)	
Ascending part	1 (0.1%)	0 (0%)	
Circumferential orientation, *n* (%)			
Anterior	200 (20.3%)	16 (25.8%)	
External	324 (32.9%)	27 (43.6%)	
Posterior	241 (24.4%)	14 (22.6%)	
Internal	221 (22.4%)	5 (8.1%)	
Lesion size, mean (SD), mm	20.8 (13.6)	28.5 (19.7)	< 0.01*
Circumferential extent, 50% or more, *n* (%)	53 (5.4%)	12 (19.3%)	< 0.01*
Macroscopic type, depressed, *n* (%)	252 (25.6%)	14 (22.6%)	0.60
Final pathology, *n* (%)			
Adenoma	423 (42.9%)	11 (17.7%)	
Intramucosal carcinoma	514 (52.1%)	47 (75.8%)	
Submucosal carcinoma	28 (2.8%)	3 (4.8%)	
Other	21 (2.1%)	1 (1.6%)	

* Statistically significant difference (*p* < 0.05).

### 3.3 Short‐Term Outcomes of Procedures

**FIGURE 2:**
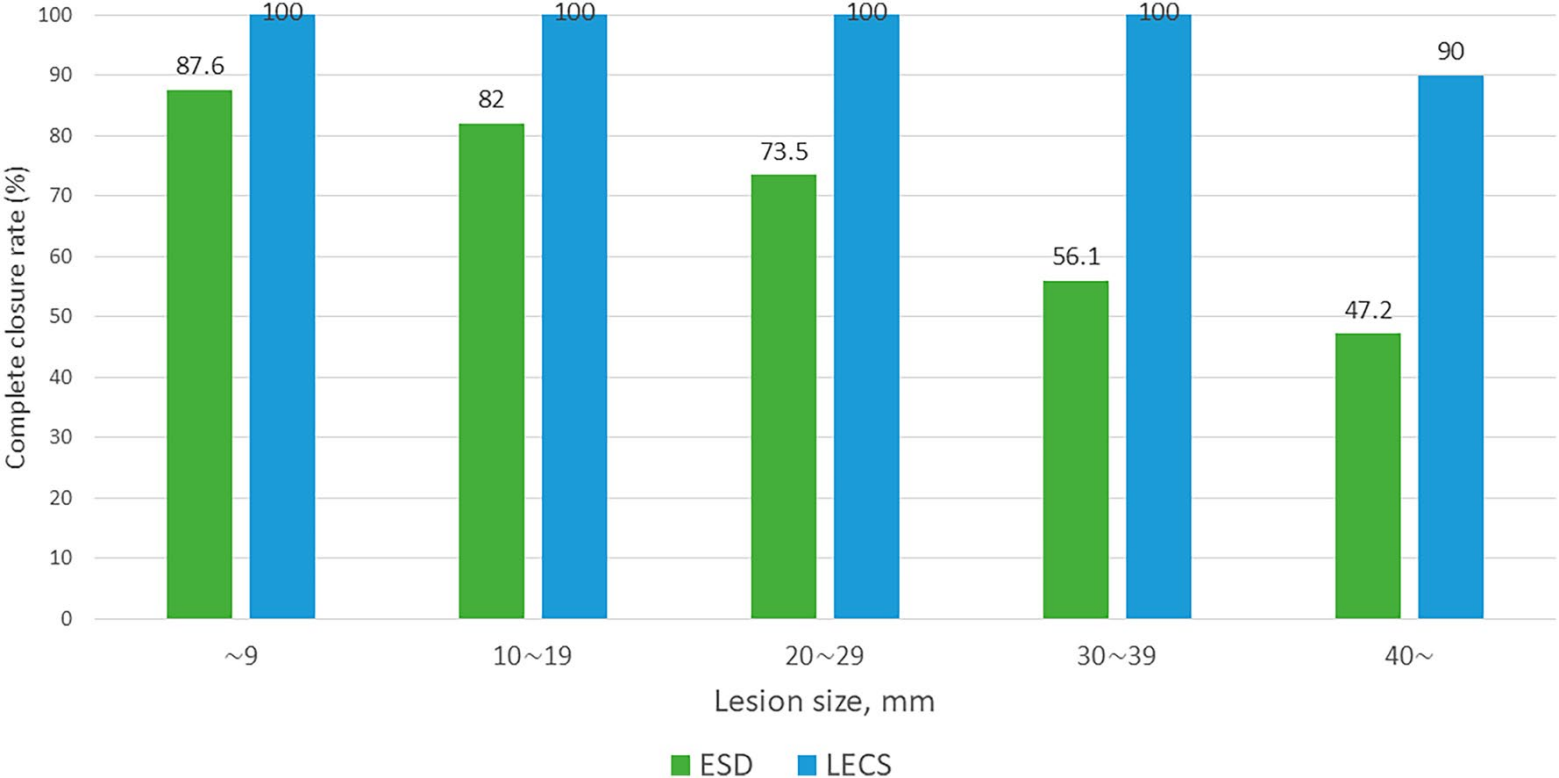
Complete closure rate of the resected wounds. ESD, endoscopic submucosal dissection; LECS, laparoscopic and endoscopic cooperative surgery.

The surgical and postsurgical outcomes of the two groups are shown in Table 2. The median (interquartile range [IQR]) procedure time in the ESD group was 58 (30–104) minutes, while that in the LECS group was significantly longer, at 206 (72–293) minutes. The rates of *en bloc* and R0 resection were similar in both groups. However, LECS achieved a significantly higher complete closure rate of the resected wounds (98.4% vs. 74.4%, *p* < 0.01). Although the complete closure rate of the resected wounds decreased with increasing lesion size in the ESD group, it was consistently high regardless of lesion size in the LECS group (Figure 2). Among the 62 patients who underwent LECS, only one case resulted in incomplete closure. This case involved both intraoperative perforation and bleeding that was difficult to control, and the procedure was performed during the early phase of the study period. Among the delayed AEs, delayed bleeding and perforation occurred in 49 (5.0%) and 22 (2.2%) patients in the ESD group and 3 (4.8%) and 1 (1.6%) in the LECS group, respectively, which showed no significant difference between the two groups. Other delayed AEs were also more frequent in the LECS group. Four patients developed acute pancreatitis, two developed aspiration pneumonia, one developed catheter‐related bacteremia, and one developed localized peritonitis in the ESD group, whereas three patients in the LECS group developed stenosis, one developed abscess formation, and one developed fluid retention with prolonged inflammation. Surgical intervention was required in 18 (1.8%) and 1 (1.6%) patients in the ESD and LECS groups, respectively. The median (IQR) hospitalization period was 8 [6, 7, 8, 9, 10, 11] days in the ESD group, which was significantly shorter than that (11 [9, 10, 11, 12, 13] days) in the LECS group (*p* < 0.01).

**TABLE 2:** Surgical and postsurgical outcomes.

	ESD (*n* = 986)	LECS (*n* = 62)	*p*
Procedure time, median (IQR), minutes	58 (30–104)	206 (72–293)	< 0.01*
*en bloc* resection, *n* (%)	935 (94.8%)	59 (95.2%)	0.91
R0 resection, *n* (%)	764 (78.4%)	50 (82.0%)	0.51
Complete closure of the wound, *n* (%)	734 (74.4%)	61 (98.4%)	< 0.01*
Delayed bleeding, *n* (%)	49 (5.0%)	3 (4.8%)	0.96
Delayed perforation, *n* (%)	22 (2.2%)	1 (1.6%)	0.75
Other adverse events, *n* (%)	8 (0.8%)	5 (8.1%)	< 0.01*
Surgery due to adverse event, *n* (%)	18 (1.8%)	1 (1.6%)	0.90
Treatment‐related death, *n* (%)	1 (0.1%)	0 (0%)	0.80
Hospitalization period, Median (IQR), days	8 (6–11)	11 (9–13)	< 0.01*

* Statistically significant difference (*p* < 0.05).

### 3.4 Risk Factors for AEs

Analyses were performed by combining the ESD and LECS groups. Multivariate analyses found that incomplete closure of resected wounds was the only independent risk factor for delayed bleeding (odds ratio [OR]: 5.069, 95% confidence interval [CI] 2.647–9.708) and delayed perforation (OR: 5.413, 95% CI 2.080–14.09). Conversely, the treatment method and ≥ 50% circumferential extent were not significant risk factors in this cohort (Tables 3 and 4).

**TABLE 3:** Logistic regression analysis for risk factors of delayed bleeding, multivariate.

	Odds ratio	95% (CI)	*p*
Age (71 or more/70 or less)	0.499	0.225–1.108	0.0878
Location (oral/anal)	0.823	0.453–1.494	0.5217
Circumferential orientation (external wall/others)	1.577	0.865–2.876	0.1370
Lesion size (20 mm or more/19 mm or less)	0.672	0.347–1.300	0.2380
Circumferential extent (50% or more/49% or less)	1.429	0.499–4.090	0.5063
Macroscopic type (depressed/elevated)	1.047	0.505–2.170	0.9028
Treatment method (ESD/LECS)	0.614	0.165–2.283	0.4664
Procedure time (121 min or more/120 min or less)	1.210	0.596–2.456	0.5981
Degree of closure (incomplete/complete)	5.069	2.647–9.708	< 0.0001*

*Note:* Location (oral): Bulbs, Supraduodenal angle, Descending part oral. Location (anal): Descending part anal, Infraduodenal angle, Horizontal part, Ascending part. Circumferential orientation (others): Anterior wall, Posterior wall, Internal wall.

* Statistically significant difference (*p* < 0.05).

**TABLE 4:** Logistic regression analysis for risk factors of delayed perforation, multivariate.

	Odds ratio	95% (CI)	*p*
Age (71 or more/70 or less)	0.801	0.283–2.264	0.6760
Location (oral/anal)	1.119	0.465–2.694	0.8027
Circumferential orientation (external wall/others)	1.848	0.769–4.441	0.1697
Lesion size (20 mm or more/19 mm or less)	1.040	0.389–2.779	0.9384
Circumferential extent (50% or more/49% or less)	1.110	0.228–5.412	0.8971
Macroscopic type (depressed/elevated)	2.498	0.924–6.753	0.0713
Treatment method (ESD/LECS)	0.919	0.106–7.960	0.9392
Procedure time (121 min or more/120 min or less)	1.310	0.474–3.621	0.6029
Degree of closure (incomplete/complete)	5.413	2.080–14.09	0.0005*

* Statistically significant difference (*p* < 0.05).

## 4 Discussion

The incidence of SDETs is extremely low, and evidence‐based therapeutic strategies have not yet been established. Several previous studies have demonstrated that less invasive treatment options, such as ESD and LECS, are oncologically acceptable for SDETs. However, most reports on SDETs have involved only a small number of patients from a single institution. Therefore, appropriate treatment options for SDETs remain unclear. Against this background, we conducted a multicenter retrospective study on the treatment outcomes for SDETs to accumulate a large number of cases, even though this is a rare disease.

As expected, lesion size was significantly larger in the LECS group than in the ESD group, and the proportion of lesions greater than half of the circumference was also higher. These results may be due to the preoperative judgment that ESD is likely to result in incomplete closure for these tumors. However, the complete closure rate of LECS was significantly higher than that of ESD.

Concerning the evaluations of the treatments, there was no difference in the *en bloc* and R0 resection rates between the two groups. This could be because the majority of tumor resections were performed using endoscopic techniques, primarily ESD, even in the LECS group. In this study, en bloc resection was achieved in over 90% of cases via ESD, regardless of lesion size or location. However, the R0 resection rate was approximately 80% even in the ESD group. These findings can be attributed to the technical difficulty of duodenal ESD and the unique anatomical characteristics of the duodenum. Indeed, because ESD is technically difficult, we maintained a small horizontal margin. In addition, the duodenal mucosa is considerably thinner than in other parts of the gastrointestinal tract, making thermal injury more likely to extend to the margins of the lesion. This increases the difficulty of accurately evaluating both the horizontal and vertical margins. Nevertheless, achieving en bloc resection remains clinically valuable, as it enables precise histopathological assessment.

Previous studies have reported that ESD for SDETs results in a high rate of AEs [7, 8, 9, 10, 11, 12, 13, 14]. Delayed perforation, which can occasionally lead to fatal conditions and requires additional extensive surgery, should be prevented. Delayed bleeding is another AE that should be prevented, although the rate of additional surgical intervention may be relatively low. To prevent or reduce these AEs, which could diminish the benefits of less invasive treatments, LECS has been introduced in the treatment of SDETs. However, the rate of delayed perforation was 2.2% in the ESD group and 1.6% in the LECS group, showing no significant reduction in the present study. There was no significant difference in the rate of delayed bleeding (5.0% in the ESD group and 4.8% in the LECS group). Notably, the frequency of delayed AEs was almost constant regardless of lesion size in the ESD group. Although the incidence of delayed bleeding and perforation did not differ significantly between the two groups, stenosis and abscess formation were observed in the LECS group. These AEs might have been associated with the laparoscopic surgical manipulation required during the LECS procedure and could have led to prolonged hospitalization.

Multivariate analyses clearly demonstrated that the treatment method was not a significant risk factor for severe AEs; however, incomplete closure of the resected wounds was a significant risk factor. These findings indicate that the most important thing to prevent such severe AEs is not which treatment options should be selected, but rather the complete closure of resected wounds using any method. Although the rate of complete closure decreased as the tumor size increased in the ESD group, complete closure was achieved in almost all patients in the LECS group, suggesting that LECS may be more useful for large (> 30 mm) tumors. However, various endoscopic closure techniques have been developed recently [25, 26, 27, 28, 29, 30, 31, 32], and if these procedures are widely adopted, the situation may change. In LECS cases, wound closure was primarily performed laparoscopically. However, for lesions located on the pancreatic side, laparoscopic closure was often technically difficult. In such situations, laparoscopic closure of the nonpancreatic side helped reduce the size of the mucosal defect, which subsequently facilitated intraluminal closure using clips. This combined approach contributed to achieving complete wound closure even in technically challenging cases.

Our study had some limitations. First, this was a retrospective cohort study. However, prospective studies are difficult to conduct for rare diseases such as SDETs. Therefore, this retrospective large cohort study is informative for discussing adequate treatment strategies for SDETs. Second, the therapeutic strategy differed among institutions, which may have affected the results of this study. Third, the relatively small number of patients in the LECS group may limit the statistical power for detecting differences in rare AEs. This limitation should be taken into account when interpreting the regression results. Fourth, the endoscopic and laparoscopic procedures were performed by highly skilled and experienced endoscopists and surgeons at each high‐volume institution. Therefore, our data cannot be standardized for low‐ or moderate‐volume institutions. This study is limited by its retrospective nature and the potential for selection bias due to differences in tumor size and characteristics between the ESD and LECS groups. Although propensity score matching was considered, it was not performed because of the relatively small number of LECS cases, which would have limited the statistical power. Future prospective or matched cohort studies are warranted to validate our findings.

In conclusion, our retrospective multicenter cohort study clearly demonstrated that both ESD and LECS showed similar AEs rates, although LECS was likely to be indicated for larger and wider circumferential lesions, and that only incomplete closure of resected wounds was an independent risk factor for severe AEs, such as delayed perforation and bleeding.

## Author Contributions

**Takuo Takehana:** conceptualization; investigation; methodology; formal analysis; writing – original draft preparation; writing – review and editing. **Tsuneo Oyama:** supervision; investigation; writing – review and editing. **Motohiko Kato:** conceptualization; supervision; investigation; writing – review and editing. **Shunsuke Yoshii:** investigation; writing – review and editing. **Shu Hoteya:** investigation; writing – review and editing. **Satoru Nonaka:** investigation; writing – review and editing. **Shoichi Yoshimizu:** investigation; writing – review and editing. **Masao Yoshida:** investigation; writing – review and editing. **Ken Ohata:** investigation; writing – review and editing. **Hironori Yamamoto:** investigation; writing – review and editing. **Yuko Hara:** investigation; writing – review and editing. **Shigetsugu Tsuji:** investigation; writing – review and editing. **Osamu Dohi:** investigation; writing – review and editing. **Yasushi Yamasaki:** investigation; writing – review and editing. **Hiroya Ueyama:** investigation; writing – review and editing. **Koichi Kurahara:** investigation; writing – review and editing. **Tomoaki Tashima:** investigation; writing – review and editing. **Nobutsugu Abe:** investigation; writing – review and editing. **Atsushi Nakayama:** investigation; writing – review and editing. **Ichiro Oda:** investigation; writing – review and editing. **Naohisa Yahagi:** conceptualization; supervision; writing – review and editing (lead).

## Disclosure

Approval of the Research Protocol: The study protocol was approved by the Institutional Review Board of Keio University School of Medicine on October 28, 2019 (approval no. 20180288), and then by each institution.

Registry and the Registration No. of the Study/Trial: N/A.

## Consent

Informed consent was obtained in an opt‐out manner on the website of each institution.

## Conflicts of Interest

The authors declare no conflicts of interest.
